# The Use of Social Networking Sites in Mental Health Interventions for Young People: Systematic Review

**DOI:** 10.2196/12244

**Published:** 2018-12-18

**Authors:** Brad Ridout, Andrew Campbell

**Affiliations:** 1 Cyberpsychology Research Group Faculty of Health Sciences The University of Sydney Sydney Australia

**Keywords:** social media, social networking, mental health, social support, support groups

## Abstract

**Background:**

The onset of mental health problems peaks between adolescence and young adulthood; however, young people face barriers to treatment and are often reluctant to seek professional help. Many are instead seeking support and information regarding their mental health via the Web, especially via social networking sites (SNSs), and hence, there is a promising opportunity to use SNSs to deliver or integrate with youth-focused online mental health interventions. Previous reviews have evaluated the effectiveness of SNSs for specific disorders in young people; however, none of the reviews have covered the breadth of SNS–based youth mental health interventions available across all mental health issues.

**Objective:**

This review aimed to systematically identify available evidence regarding the use of SNS–based interventions to support the mental health of young people aged up to 25 years, to evaluate their effectiveness, suitability, and safety, and identify gaps and opportunities for future research.

**Methods:**

The PubMed and PsycINFO databases were searched using Medical Subject Headings terms and exploded keywords and phrases. Retrieved abstracts (n=974) were double screened, yielding 235 articles for screening at the full-text level. Of these, 9 articles met the review inclusion criteria. Given the small number of studies, and the variety of outcome measures used, a quantitative meta-analysis was not possible.

**Results:**

The 9 articles (quantitative studies, qualitative studies, and descriptions of the iterative design process) covered 5 separate interventions. Of the 5 interventions, 2 interventions used purpose-built platforms based on the moderated online social therapy (MOST) model, 2 used Facebook, and 1 evaluated a purpose-built mobile app. The 2 MOST interventions targeted specific mental health issues (depression and psychosis), whereas the others focused on improving mental health literacy, social support, and general well-being. Only 3 quantitative studies were identified, and all used a pre-post design (without a control group) to establish *proof of concept*. Of the outcome variables assessed, there were significant improvements in mental health knowledge and number of depressive symptoms but no improvement in anxiety or psychosis symptoms. Acceptability of and engagement with the SNS platforms were generally high, as were perceptions of usefulness and safety. Moderation by clinical experts was identified as a key component of the more successful interventions. When offered a choice, users showed a preference for mobile apps over Web-based interfaces.

**Conclusions:**

The evidence reviewed suggests young people find SNS–based interventions highly usable, engaging, and supportive. However, future studies need to address the current lack of high-quality evidence for their efficacy in reducing mental health symptoms. Given young people are already turning to SNSs to engage in knowledge seeking and peer-to-peer support, SNS–based youth mental health interventions provide an opportunity to address some of the barriers young people face in accessing qualified mental health support and information.

## Introduction

Supporting the mental health of young people is a major public health challenge, with mental disorders accounting for almost half of the nonfatal burden of disease among people aged 10 to 25 years [1]. Adolescence is a particularly vulnerable period of development, with the onset of mental health problems peaking between adolescence and young adulthood [2]. However, many problems are not detected until later in life, as young people are often reluctant to seek professional help [3] and face barriers to treatment such as cost, poor mental health literacy, confidentiality concerns, stigma, and inaccessibility to or lack of knowledge of resources [4,5].

Given that internet-enabled mobile devices have become a near-ubiquitous element of adolescence, with 45% of teens admitting that they are online *almost constantly* [6], it is not surprising that young people are increasingly seeking support and information regarding their mental health online [7].

Over the past decade, social media has become an important element of communication for young people, with virtually all having at least one active social media account [6]. People with mental illness are often among the highest users [8], with many reporting that social media fosters community among users and makes them feel supported and accepted [9]. Furthermore, a recent study found that actively engaging with peers online about their mental health concerns was associated with an increased likelihood of seeking formal mental health care [10].

Social networking sites (SNSs), a subset of social media, have become the predominant context for communication and social support–seeking behaviors online among adolescents [11]. SNS users create a profile within a bounded system, which they use to make and display connections with other users [12]. Posting of user-generated and Web-based content and functions such as liking, commenting, and tagging are the lifeblood of SNSs and differentiate SNSs from Web 1.0 communication tools such as message boards and online support groups [13].

Given the barriers to mental health support young people face and the fact that they are naturally turning to SNSs to engage in knowledge seeking and peer-to-peer support, there is a promising opportunity to use SNSs to deliver or integrate with youth-focused online mental health interventions. Compared with other online mental health resources such as online counseling, mobile apps, and online support groups, research into the use of SNSs to support and treat young people with mental health issues is only in its infancy and is highly fragmented. Although there have been reviews evaluating the effectiveness of SNSs for specific mental health disorders in young people [14,15] and online peer-to-peer support for young people more broadly [16], none of the reviews have covered the breadth of SNS–based youth mental health interventions available across all mental health issues. A systematic review of the literature regarding the use of SNS–based interventions to support the mental health of young people is, therefore, required to evaluate their effectiveness, suitability, and safety and identify gaps and opportunities for future research.

## Methods

### Search Strategy

This systematic review was performed using the preferred reporting items for systematic reviews and meta-analyses (PRISMA) guidelines [17]. A PRISMA checklist is available in Multimedia Appendix 1. PubMed was searched using Medical Subject Headings terms, and PsycINFO was searched using exploded keywords and phrases (see Multimedia Appendix 2). Searches were conducted in June 2018 and restricted to English-language articles published in peer-reviewed journals between January 2000 and June 2018.

In total, the database searches yielded 1020 records (592 from PubMed and 428 from PsycINFO), of which 60 duplicates were removed. Additionally, 14 records were identified through manual searches of previous reviews, key journals, and reference lists of key articles.

### Screening Process

**Figure 1:**
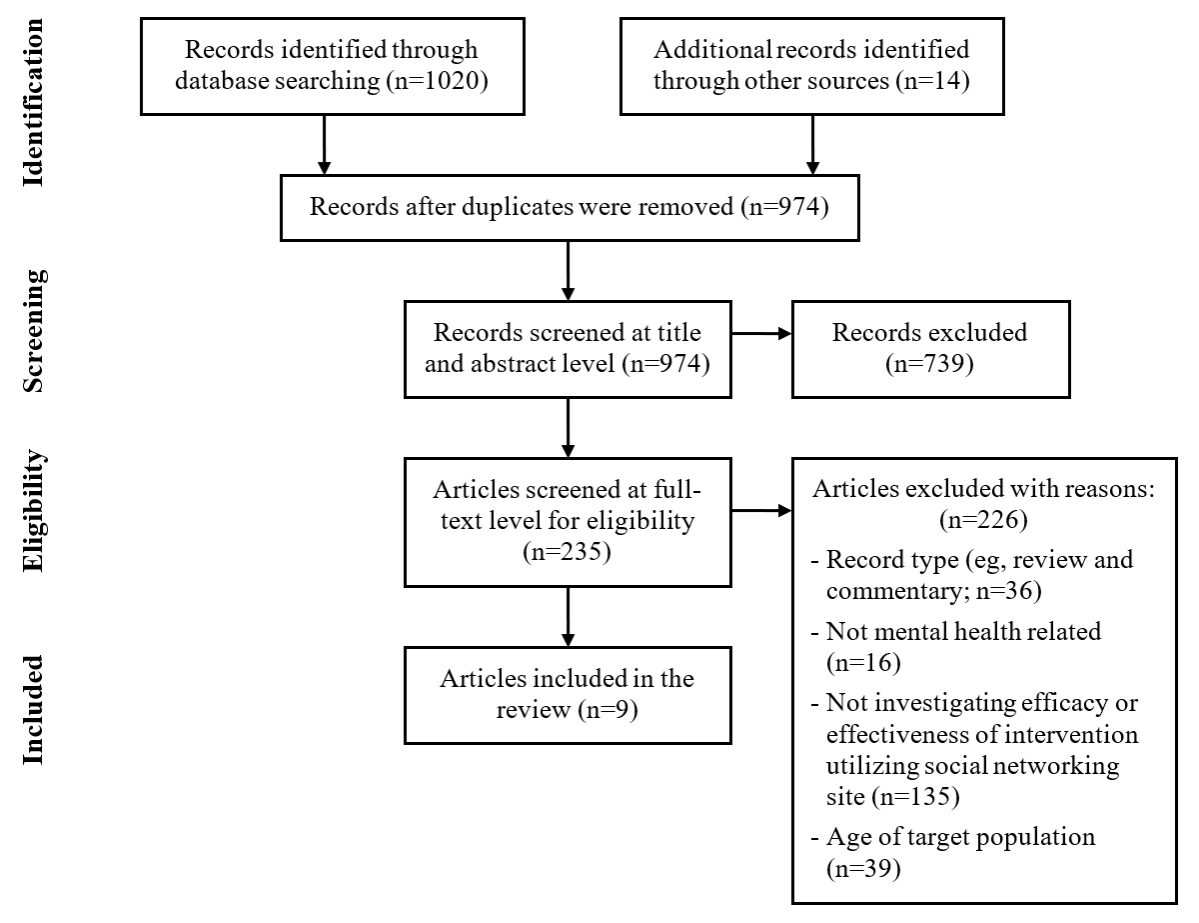
PRISMA flow diagram.

Figure 1 presents a PRISMA flow diagram of the screening process, which involved 2 stages: (1) title and abstract exclusion and (2) full-text exclusion. All records were independently screened by 2 reviewers (BR and AC) to establish relevance for inclusion at both stages. Any discrepancies between the reviewers were resolved by discussion. Of the 974 records identified (after duplicates were removed), 739 were removed because their titles and abstracts indicated they were not relevant to the topic of using SNS to support youth mental health. This left 235 articles to be assessed for eligibility according to predefined inclusion criteria.

The inclusion criteria were as follows: (1) the record must be an original empirical study (ie, not a review or commentary), (2) the primary aim of the study must be to address either a specific mental health condition or improve mental health and well-being generally, (3) the study must investigate the efficacy or effectiveness of a specific intervention utilizing an SNS (as defined by Boyd and Ellison [12]) to improve youth mental health (ie, not the impact of naturally occurring SNS support groups), and (4) the target population of the intervention must be young people aged up to 25 years.

A total of 36 articles were excluded during the second screening stage based on record type (eg, review and commentary) and 16 were excluded because they were not mental health related. A total of 135 articles were excluded as they were not investigating the efficacy or effectiveness of an intervention utilizing an SNS to improve youth mental health, and 39 articles were excluded based on the age of the target population being other than young people aged up to 25 years.

### Data Analysis

Given the small number of studies included in this review, their exploratory nature, and the variety of outcome measures used, a quantitative meta-analysis was not possible. Primary and secondary outcome measures related to mental health are, therefore, reported (with effect sizes where possible) along with the characteristics of and usability and engagement data regarding the social networking components of the interventions.

## Results

### Study Characteristics

Detailed characteristics of the included articles (n=9) are provided in Multimedia Appendix 3. Of the 9 articles, 3 articles reported uncontrolled pilot studies that utilized a pre-post design [18-20] and 4 reported qualitative evaluations (2 of these evaluating 1 of the aforementioned pilot studies each) [21-24]. The 2 remaining articles were descriptions of the iterative design process of 2 of the already included studies [25,26]. In summary, there were 5 separate studies covered by the 9 included articles.

The articles were categorized according to the mental health issue they were focused on: psychotic disorder or mood disorder with psychotic features (n=3) [18,21,25], depression (n=2) [19,22], and health literacy and well-being (n=4) [20,23,24,27].

### Origin

A total of 7 articles were from Australia, and the remaining articles were from the United States and Hong Kong. There were many studies from the United States and Europe included at the first screening stage; however, the majority of those that were focused on youth mental health were excluded at the second screening stage because they were investigating the impact of naturally occurring SNS groups, rather than purpose-built interventions for supporting youth mental health (an area that Australia is currently pioneering).

### Interventions

Overall, 3 separate purpose-built SNSs were the focus of 7 of the articles (Horyzons: 3, Rebound: 2, and MindMax: 2), whereas the remaining articles evaluated interventions that used Facebook (one using a purpose-built Facebook game and the other using a closed Facebook group). Both Horyzons and Rebound were based on the moderated online social therapy (MOST) model developed by members of their research team [26], whereas MindMax took a modular approach combining well-being science, video games, and personal experience and stories of professional Australian Football League (AFL) players. Of the purpose-built apps, only MindMax was made available to the public.

### Participants

Most of the samples consisted exclusively of participants with self-reported mental health concerns relevant to the focus of the respective study (n=5). Of the remaining articles, 2 articles used nonclinical samples of university students, and the 2 articles investigating the design of MindMax used convenience samples from the target audience of the app. As per the inclusion criteria, all studies aimed to support the mental health of children and/or young people, with the youngest participants across the studies being aged 15 years. The mean age of participants for all studies fell within a range of 18 to 21 years, except for the qualitative evaluation of MindMax [23], which was based on focus interviews of 7 participants (6 males and 1 female) with an age range of 24 to 49 years (average of 35 years). Although the convenience sample for this initial usability study was mostly made up of participants aged older than 25 years, it was included in this review as the target population of the MindMax app is young people aged 16 to 25 years. Gender was relatively balanced in all other studies (42%-50% male), except for the YBMen study, which focused on students who identified as black men. Participants were recruited from a range of sources and methods. The Horyzons and Rebound studies recruited from early intervention clinics, the Facebook studies from an online network of university students, and the MindMax studies were based on participatory design workshops and focus interviews with AFL fans, players, gamers, mental health and well-being consumers, clinicians, researchers, and academics.

### Outcome Measures

The Horyzons study focused on mood disorders with psychotic features and used the Brief Psychiatric Rating Scale, the Calgary Depression Scale for Schizophrenia, and the Beck Anxiety Inventory as primary outcome measures to determine reduction in symptoms of psychosis, depression, and anxiety, respectively. The Rebound study focused on depression used the interviewer-rated Montgomery-Asberg Depression Rating Scale (MADRS) as its primary outcome measure, along with other secondary measures of anxiety, social and occupational functioning, strengths use, social support, and social connectedness. The Facebook game study focused on mental health literacy used a self-assessment questionnaire developed by the researchers to assess this primary outcome and modified questions from the Motivated Strategies for Learning Questionnaire to assess learning motivation as the secondary outcome. The authors of the closed Facebook group study, which also focused on improving mental health literacy (in addition to providing social support), mentioned that quantitative outcome measures were collected; however, only results of the qualitative interviews have been published to date. The MindMax study used participatory design workshops and focus interviews to evaluate usability and initial experiences of using the app, whereas outcomes related to the app’s main aim to improve mental health literacy and well-being were being studied at the time of this review.

### Study Quality

As all studies included in this systematic review were uncontrolled pilots or exploratory studies of acceptability or usability, no formal assessment of quality was performed. All 3 of the quantitative articles included used a pre-post design without a control group and aimed to provide *proof of concept*, rather than causal inferences about efficacy or effectiveness. The 2 MOST studies reported using completer analyses only (one of these had no dropouts and the other reported a dropout rate of 7.1%). The Facebook game study had a dropout rate of 42.5% and used multiple imputations to address loss of follow-up data for the 54 dropouts so that they could be included in an intention-to-treat (ITT) analysis. The result of the ITT analysis was consistent with the completer analysis conducted, which used only the 73 participants who completed both the pre- and posttest questionnaires.

The qualitative articles for the 2 MOST studies conducted semistructured interviews at the conclusion of the trial only, whereas the MindMax and closed Facebook group studies conducted structured interviews at multiple time points throughout the trial (although the latter only reported results of interviews conducted postintervention).

All 4 qualitative studies transcribed the interviews and coded them for thematic analysis using either QSR NVivo software or spreadsheet techniques. The Rebound and MindMax studies followed the established thematic analysis guidelines of Braun and Clarke [28], whereas the Horyzons study followed accepted qualitative methods [29,30] that recommend conducting multiple parses of the qualitative data with different levels of coding. The closed Facebook group study applied a data reduction technique developed by the lead author called the *rigorous and accelerated data reduction* technique [31].

### Characteristics of Social Networking Functions

The social networking environment of the Horyzons and Rebound platforms was known as *The Café*. The platforms included a newsfeed where participants and moderators could post text, pictures, and videos and *like* or comment on posts of other users, similar to well-known Facebook functions. The newsfeed incorporated categories to organize discussion threads into themes (eg, *what’s on your mind*, *I’m loving right now*, *cheer me up*, and *strength news*). The system also included a *homepage*, showing all the activity and notifications relevant to the participant. Participants could also view the *wall* of others, displaying that participant’s individual activity (similar to what Facebook now refers to as the *timeline*), and their own *network* (similar to Facebook’s *friends* function).

On the basis of the MOST model [26], the Horyzons and Rebound platforms were designed to reinforce the therapeutic content of the interventions and ensure constant flow between the therapy and social networking components. This was achieved by integrating questions within the therapeutic content to promote discussion and encourage users to share their own experiences, which then become discussion threads within The Café. Both platforms also featured an online group–based problem-solving space (known as *Talk It Out* in Rebound), guided by moderators within the social networking environment. Using an evidence-based problem-solving framework [32,33], moderators guided participants through the structured phases of problem definition, brainstorming solutions, identifying pros and cons, and summarizing possible choices. Offered solutions and participants’ experiences were then saved in a database for participants to refer to throughout the intervention. The Horyzons intervention also included a *job zone*, where users could access information regarding training and vocational recovery, or *ask Gina*, an expert in this area.

The MindMax platform also featured a familiar Facebook-style interface, with the main social networking function being a newsfeed where participants could post text, pictures, and animated graphics interchange formats (but not videos) and *like* or comment on posts of other users. Participants could also use hashtags to make their posts searchable to other participants (eg, #gratitude, #values, and #fitminds) or share their MindMax posts on other SNS such as Facebook. Integrated alongside the newsfeed were the *Train* well-being education modules (*Fit Minds*, *Values*, and *Thoughts*) and *Play*, where participants could use the *footies* awarded for completed activities within the education modules to play the *Flick Footy* video game and then post their score in the newsfeed for other participants to view, *like*, and comment on. There was also a *Me* function where participants could update their profile and view their activities and saved posts. The *Train* modules often included text and videos from AFL players and short questionnaires and activities. Completed activities were automatically posted to the newsfeed with appropriate hashtags by default (this could be turned off in profile settings). Being a mobile phone app, MindMax utilized push notifications to alert users about activities relevant to them when not using the app.

The purpose-built game *Ching Ching Story* was an app within Facebook, and as such, its social networking functions interacted with those of Facebook. For example, *task completed* notifications were posted on players’ Facebook walls (now known as *timelines*) to acknowledge achievements in the game and encourage interaction between players via Facebook *likes* and comments. Additional functions within the game itself included the ability to send friends greetings, gifts, and special *tools* needed to accomplish certain tasks. Gifts were also offered to players for sending invitations to their Facebook friends to join the game. A leaderboard was also available within the game to create an atmosphere of competition.

The other Facebook study, YBMen, used a closed Facebook group to post educational material (taken from gender and culturally relevant popular culture references) and daily prompts for group discussion about the importance of mental health, social support, and the challenges associated with rigid adherence to masculine norms. Participants communicated with each other and the study team using standard Facebook functions (comments, *likes*, and posting and sharing content). Group facilitation techniques included group problem solving, action planning and feedback, and individual decision making to improve mental health behaviors and outcomes.

### Moderation

The inclusion of expert moderators with clinical experience was a key feature of the MOST platforms. The moderators in Horyzons were clinical psychologists and vocational workers, identified within the social networking platform as *coach*. They moderated the site daily for 1 to 2 hours, and their role was to “guide, but not censor, the interaction to ensure a safe and supportive environment” [18]. The moderators in Rebound were experienced youth mental health clinicians who monitored the site daily. In addition, both platforms used an auto-detect risk management system to identify keywords associated with risk of relapse, self-harm, or suicide, which would then trigger crisis protocol and risk assessment.

The Rebound study also featured peer moderators known as *Super Users* —young people with recent lived experience of mental illness who were given training and supervision to provide peer support to other users of the site. However, most users were not aware of the Super Users (identified only by a distinct symbol on their avatar) and, therefore, did not recall interacting with them. Those who were aware of Super Users thought that they were useful as role models and gave them hope that they could also recover. The previously conducted Horyzon study did not include peer moderators; however, most users believed that including previous users of the site as peer moderators would be beneficial, and 90% reported interest in becoming one themselves.

The moderator in the YBMen Project was the lead author, an African American female researcher with 13 years’ experience in research and community interventions on the mental health of black men. The moderator and her team (male and female graduate students) were responsible for not only monitoring the site but also for posting the daily educational material along with questions to generate group discussion and facilitate engagement, in contrast to the MOST platforms where therapeutic content was included in modules for users to work through at their own pace. It was not clear whether the YBMen moderator and her team were individually identifiable with the Facebook group or whether they all used the same Facebook user account.

The MindMax and Ching Ching Story studies did not report whether moderation was a feature of their platform.

### Intervention Efficacy

#### Depression, Anxiety, and Psychosis Symptoms

A moderate to large reduction in participants’ depressive symptoms (*d*=0.6) was found after using Horyzons for 1 month [18]. A small reduction in anxiety symptoms was found but failed to reach significance, and there was no reduction in psychosis symptoms between pre- and postintervention.

A similar effect size for reduction in depressive symptoms was reported by Rice et al [19] in their pilot study of Rebound, with a significant improvement in interviewer-rated depression scores on the MADRS after 2 months (*d*=0.45). There was no improvement in anxiety, social and occupational functioning, social support, or social connectedness; however, there was a trend (*P*<.1; *d*=0.29) for improved strength use.

#### Mental Health Literacy

The study measuring health literacy demonstrated a moderate to large improvement in performance on their 31-question knowledge test (*d*=0.65) following a 3-week period of using the purpose-built Facebook game [20]. Intrinsic goal orientation was identified as the primary factor in learning motivation. Self-efficacy for learning and performance significantly predicted learning outcomes, whereas test anxiety was found to be negatively associated with learning outcomes. The MindMax and closed Facebook group studies also aimed to improve mental health literacy; however, studies published to date have not reported this as an outcome variable.

### Engagement

User engagement data for the Horyzons platform suggested high use among participants, with 60% using the system in each of the 4 weeks (70% in at least 3 out of 4 weeks). The social networking component was used by 95% of participants, with a median of 192 social page views/actions per participant. Module use varied across participants; however, 95% completed at least one full therapy module and 60% completed at least three modules (of 7 available). There was a median of 65 therapy-based page views per participant.

Engagement with the Rebound platform was also high, with 70% logging in weekly (78.5% in at least 2 of 3 months). The social networking component was used by all participants, with a mean of 51.1 social posts per participant. In terms of therapeutic content, 42.9% completed 5 or more therapy modules (of 56 available) and 26.2% reported completing 5 or more *actions* (applying therapy content in the offline world).

The structured interviews conducted to investigate initial experiences with the MindMax platform revealed that participants found the gaming and sporting elements to be the most engaging aspects of the app, with many returning to use the game even after completing all the well-being training. Statistics of engagement with the social networking functions are anticipated to be reported in a future report of the currently ongoing evaluation trial of MindMax.

Engagement with the YBMen intervention was assessed via quantitative data collected about the level of Facebook activity recorded. Most participants (67.3%) viewed Facebook postings at least weekly and around half (50.9%) actively contributed each week by commenting or posting new material.

Assessment of engagement with the purpose-built Facebook game was not reported.

### Usability

Horyzons was considered a useful long-term treatment option beyond discharge by 70% of participants, with a majority reporting that it significantly increased their social connectedness (60%) and empowered them in their own recovery process (55%). The social networking component of Horyzons was perceived as useful by 70% of participants. Moreover, 90% of participants considered moderation to be supportive and 85% thought it would be beneficial to include peer moderators who were previous users of Horyzons (with 90% reporting they would like to become online peer moderators). There were no incidents (ie, adverse events or inappropriate usage) during the study.

User experience data for the Rebound platform were collected via a standardized industry tool for benchmarking websites called Web Analysis and Measurement Inventory (WAMMI) [34]. Rebound rated above average on all 5 WAMMI domains (attractiveness, controllability, efficiency, helpfulness, and learnability), achieving a global utility percentile rank of 59.6. In addition, ratings on a 1- to 5-point scale were collected to assess safety (4.7), helpfulness (3.6), and perceived benefits related to social connectedness (3.5). Impressions of the moderators were assessed on 1- to 7-point scales, with participants rating their agreement that moderators encouraged open discussion (6.0), accepted them (5.8), provided them with choices (5.5), and listened to how they would like to use Rebound (5.6).

Initial usability testing of MindMax with 3 users revealed an appreciation for the gamification of content and shared use by known AFL players [27]. Concerns were expressed regarding privacy and the possibility that users may only post or *like* content to get points, rather than meaningfully engage with the app.

Interviews conducted at the conclusion of the YBMen study revealed that the use of Facebook as the intervention platform was well-liked by participants, as they appreciated being able to receive notifications alerts on their mobile phones. Many participants also liked that Facebook facilitated conversations that they would not feel as comfortable having face-to-face. Barriers to engagement identified included not being able to understand some of the language used by the moderator.

Assessment of the usability of the purpose-built Facebook game was not reported.

## Discussion

### Principal Findings

The aim of this systematic review was to identify studies investigating the use of SNS to support the mental health of children and youth. A total of 9 articles reporting on 5 separate studies were identified. Of the 9 studies, 2 studies targeted specific mental health issues (depression and psychosis), whereas the other studies focused on improving mental health literacy, social support, and general well-being. Only 3 quantitative studies were identified and all used a pre-post design (without a control group) to establish *proof of concept*, rather than causal inferences about efficacy. Although this precluded any meta-analysis or assessment using Effective Practice and Organization of Care quality criteria, some of the outcome measures produced encouraging results, with significant reductions in depressive symptoms and significant improvements in mental health knowledge. However, there was no significant reduction in anxiety or psychosis symptoms. Acceptability and usability of the platforms reviewed were generally high, as were perceptions of usefulness and safety. There were no adverse incidents reported in any of the studies. When offered a choice, users showed a preference for mobile apps over Web-based interfaces and appreciated receiving notification alerts on their mobile phones. Overall, this review found evidence for the potential for SNS–based interventions to support the mental health of young people.

Engagement with the SNS platforms was high in most studies, with low dropout rates, and most users logging in and actively posting and engaging with content, moderators, and other users, on at least a weekly basis. Moderation was identified as a key component of the success of the interventions. The therapeutic interventions that were most favorably viewed by users were those that were guided by moderators within the social networking environment, with users generally finding moderators to be friendly, supportive, and caring. There was also initial support for the inclusion of peer moderators to act as role models and support the experience of other users; however, it is not suggested that these should replace the role of expert moderators with clinical experience.

Positive feedback on the benefit of giving and receiving peer-to-peer support was also received, consistent with established literature [35,36]. Users of the MOST platforms reported that the most valued characteristic of the intervention was the ability to connect with other young people of a similar age with shared experiences, backgrounds, and mental health issues. Users felt safe because the sites could only be accessed by clients of the mental health services from which they had been recruited, which also contributed to feelings of belonging to a group of peers with similar experiences. There were indications that users felt understood, supported, more socially connected, and more willing to discuss their issues as a result of interacting with peers who were facing challenges similar to them.

However, not all users were active in their use of the social networking functions, with qualitative feedback revealing that some users preferred to *eavesdrop* on discussions taking place or *lurk*. Santesteban-Echarri et al [22] identified 2 clear subgroups of low interactors in the Rebound study. The first subgroup did not like online interaction and/or had sufficient offline support, supporting the typology of social media users by Fergie et al [37] that suggests the more offline support someone has, the less regularly they engage with health-related content on social media. The second subgroup of low interactors simply felt too shy, indicating that either the activity on the site was not high enough for them to feel comfortable to initiate a conversation or that not *knowing* fellow users was a barrier (despite anonymity being one of the obvious advantages and aims of closed SNSs for supporting youth mental health). This desire to know other users was also raised by participants in the YBMen project, who suggested that having occasional face-to-face meetings would have benefited the intervention (although the authors note that this may have been influenced by the project’s association with an existing offline group).

Although it is possible that having a less than positive regard for anonymous online interactions may be a potential barrier to gaining benefit from SNS–based youth mental health interventions, more research is needed to establish whether eavesdropping on discussions may still be beneficial for low interactors. Recent research suggests that having a strong sense of community and inclusive culture are important factors for deriving positive outcomes among lurkers of online health support groups [38,39]. The design of SNSs for supporting youth mental health should, therefore, engage in strategies to create a sense of community and promote regular contributions from users [40], given it appears that variable levels of interactivity and engagement over time are features of these platforms.

Overall, the integration of the social networking components with the psychoeducation and therapy modules in the MOST interventions was considered successful, as evidenced by a high level of engagement with both and positive qualitative feedback from users. The MindMax and Ching Ching Story platforms also aimed to integrate social networking functions with the online education activities around mental health literacy, for example, by encouraging users to post about their successes in completing activities and comment on the successes of others. However, there was no evidence provided to suggest that the social networking functions were well utilized during the trials of these 2 platforms (although it was stated that *social connectedness* of MindMax users will be reported in a future evaluation of a naturalistic trial).

The need to integrate therapeutic and social networking functions was not an issue for the YBMen project, as all activities took place within the closed Facebook group. This had the additional benefit of locating the intervention within a platform that most users were already familiar with and using daily on multiple devices, including mobile devices, which was something that users appreciated. Although using naturally occurring SNS such as Facebook to deliver interventions could be a way to address the difficulty that purpose-built platforms may face in creating the norms, dynamics, and atmosphere of naturally occurring online communities [41], more evaluation is needed regarding the potential benefits and risks of using such widely used SNSs for this purpose [42].

In their commentary on the future of peer-to-peer support on social media, Naslund et al [43] identified several risks that should be considered in the design of any platform that enables peer-to-peer support. First, there are risks inherent with obtaining advice from nonexpert peers who may unwittingly pass on misleading or unreliable information. Although research shows that many users of online health forums are aware of the need to evaluate the accuracy of advice received and whether it applies to their own circumstances [44], it is not known whether young people with mental health concerns do so routinely. Second, similar to all online environments, there is the potential to be exposed to hostile or derogatory comments from others, which could have a negative impact on the mental health of users. These key risks can be largely mitigated against on closed SNSs by having clinically trained moderators regularly review posts made by users so that they can clarify, correct, or potentially remove any posts that may be problematic for other users. Although none of the studies in this review reported the need to address any problematic posts, the MOST and YBMen interventions did have this ability, as their expert moderators were actively engaged with all content posted. The presence of expert moderators greatly contributed to users’ perception of safety of the platforms.

### Limitations

It is important to acknowledge the limitations of this systematic review. First, searches were conducted in 2 databases only, limited to English-language publications, and excluded grey literature. Although the selected databases contain the largest number of health, medical, and psychological journals, this search strategy was complemented by hand searches of previous reviews, key journals, and reference lists of key articles, which yielded an additional 14 articles. Searches were current at June 2018, but as mentioned above, some of the interventions evaluated in the included articles were either ongoing or had collected additional data that were intended to be published in the future (eg, a 5-year randomized controlled trial of Horyzons was recently completed); therefore, it is likely that further articles will soon appear in scholarly journals. Finally, it is possible that this review was subject to publication bias should authors have failed to publish studies with null or negative findings.

### Conclusions and Implications for Future Research

This review updates and expands previous reviews of the use of SNS for supporting youth mental health, which have, to date, only focused on specific disorders. By broadening the scope to include all aspects of mental health, including mental health literacy, this review shows that SNSs may play a useful role in providing mental health support to both clinical and nonclinical populations. It has also highlighted the importance of involving end users across all stages of intervention and platform design development according to participatory design principles [45] and suggests that users prefer to be able to access SNS interventions on their mobile devices.

The evidence reviewed suggests that young people find SNS–based interventions highly usable, engaging, and supportive. However, high-quality evidence for their efficacy in reducing mental health symptoms is currently lacking. Furthermore, the majority of data collected in the reviewed studies came from participants aged over 18 years; therefore, there is a particular need for further investigation into the suitability of SNS–based interventions for adolescents aged less than 18 years. Now that proof-of-concept is established for some of the SNS interventions reviewed here, higher quality studies are required (ie, randomized controlled trials over longer periods), with populations that focus on adolescents as well as young adults, to build the evidence base in this field and address the following unanswered questions: Which aspects of SNS interventions are most beneficial for users and how do they mediate mental health outcomes?, Do skills gained online translate to sustained improvements in offline functioning and well-being?, Are some mental health issues and/or phases of the users’ journey better suited to SNS interventions than others?, What level of participation is required from users to gain benefit?, Are mobile apps and mobile-friendly interfaces more beneficial for users?, and Is there an optimum user group/community size? There are also methodological challenges to address such as those associated with evaluating multicomponent interventions, collecting objective measures of mental health outcomes online, and dealing with variable levels of engagement and retention over longer periods.

Overall, the evidence reviewed suggests that both clinical and nonclinical users found SNS–based interventions to be safe, engaging, supportive, and useful. When moderated, ideally by mental health professionals, the benefits of SNS–based interventions for youth mental health appear to outweigh any potential risks. Given that young people are already turning to SNSs to engage in knowledge seeking and peer-to-peer support, SNS–based youth mental health interventions present a promising opportunity to help address some of the barriers young people face in accessing qualified mental health support and information. They also provide an opportunity to combine the well-established benefits of peer-to-peer support with accessible and cost-effective online interventions.

## Appendix

Multimedia Appendix 1 PRISMA checklist.

Multimedia Appendix 2 Database search strategy.

Multimedia Appendix 3 Study characteristics.

## Abbreviations

AFL: Australian Football League
ITT: intention-to-treat
MADRS: Montgomery-Asberg Depression Rating Scale
MOST: Moderated Online Social Therapy
PRISMA: preferred reporting items for systematic reviews and meta-analyses
SNSs: social networking sites
WAMMI: Web Analysis and Measurement Inventory
